# Haploidentical haematopoietic stem cell transplantation for malignant infantile osteopetrosis and intermediate osteopetrosis: a retrospective analysis of a single centre

**DOI:** 10.1186/s13023-021-01955-6

**Published:** 2021-07-15

**Authors:** Guanghua Zhu, Ang Wei, Bin Wang, Jun Yang, Yan Yan, Kai Wang, Chenguang Jia, Yanhui Luo, Sidan Li, Xuan Zhou, Tianyou Wang, Huyong Zheng, Maoquan Qin

**Affiliations:** 1 Hematology Center, Beijing Key Laboratory of Pediatric Hematology Oncology, National Key Discipline of Pediatrics (Capital Medical University), Key Laboratory of Major Disease in Children, Ministry of Education, Beijing Children’s Hospital, Capital Medical University, National Center for Children’s Health, Beijing, 100045 China; 2 Department of Hematology and Oncology, Beijing Children’s Hospital, Capital Medical University, Nanlishi Road No. 56, Xicheng District, Beijing, 100045 China

**Keywords:** Malignant infantile osteopetrosis, Intermediate osteopetrosis, Haploidentical haematopoietic stem cell transplantation, Prognosis, Graft versus host disease

## Abstract

**Objective:**

To evaluate the clinical efficacy of haploidentical haematopoietic stem cell transplantation (haplo-HSCT) for the treatment of malignant infantile osteopetrosis (MIOP) and intermediate osteopetrosis.

**Methods:**

Children with MIOP and IOP who underwent haplo-HSCT in Beijing Children’s Hospital, Capital Medical University, from January 2010 to May 2018 were retrospectively analysed. Data relating to the clinical manifestations, engraftment, and prognosis of the children were extracted from medical records.

**Results:**

Twenty-seven patients, including 18 males and 9 females, with an onset age of 12 (0.04–72) months were enrolled in this study. The median time from diagnosis to transplantation was 4 (1–23) months. All patients received haplo-HSCT with a myeloablative conditioning regimen (including fludarabine, busulfan, and cyclophosphamide). Graft versus host disease (GVHD) prophylaxis was based on anti-human T lymphocyte porcine immunoglobulin/anti-human thymus globulin, methotrexate, and mycophenolate mofetil. The median observation time was 55.2 (0.3–126.2) months. By the end of follow-up, twenty patients survived and seven patients died. The 5 year overall survival rate was 73.9%. Stage I-II acute GVHD was observed in 20 patients, stage III GVHD in 1 patient and no patients had stage IV disease. Chronic GVHD was observed in 11 patients (40.7%) and was controlled by anti-GVHD therapy.

**Conclusions:**

Haplo-HSCT was an effective treatment for MIOP and IOP, with a high survival rate and significantly improved clinical symptoms. For patients with a vision impairment before HSCT, the improvement was slow after transplantation. The incidence of GVHD was high but mild and was effectively controlled by appropriate treatment. These data indicated that haplo-HSCT was a feasible treatment for MIOP and IOP.

## Introduction

Osteopetrosis, which is also called marble bone disease, refers to a heterogeneous group of rare inherited skeletal dysplasias. Inheritance is divided into autosomal recessive, autosomal dominant or X-linked patterns [1]. The most severe cases are almost always autosomal recessive and are termed malignant infantile osteopetrosis (MIOP). This rare inherited disease has an incidence of 1:200,000 to 1:300,000, but higher rates are reported in Russia and the Middle East [2, 3]. It is characterized by quantitative or qualitative osteoclast defects that lead to increased bone mass and density. These children present with dysplasia, hydrocephalus, fracture, hypocalcaemia, progressive bone marrow failure, neurological disorders and other conditions in the first year of life.

The prognosis of patients with MIOP is very poor, and death in the first decade is common without appropriate therapy. Haematopoietic stem cell transplantation (HSCT)is the only effective treatment for MIOP [4, 5]. Intermediate osteopetrosis (IOP) is mostly caused by the autosomal dominant inheritance of an abnormal CLCN7 gene. Some patients have earlier onset ages and may also show manifestations such as MIOP. No clear guidelines are available for transplantation in these patients [6, 7]. Here, we report the long-term survival of 27 patients who received haploid haematopoietic stem cell transplantation (haplo-HSCT) to treat MIOP and IOP in Beijing Children’s Hospital affiliated with Capital Medical University to further explore the safety and feasibility of haplo-HSCT as a treatment for osteopetrosis.

## Patients and methods

### Patients

This study employed a retrospective observational design. Children suffering from MIOP/IOP who underwent haplo-HSCT between January 2010 and May 2018 were enrolled in this study. None of the patients had an HLA-matched sibling or unrelated donors in the China Bone Marrow Bank. Data were retrospectively reviewed for the source of haematopoietic stem cells, conditioning regimen, adverse effects, and prognosis. This study was conducted in accordance with the Declaration of Helsinki and approved by the Institutional Review Board (IRB) of Beijing Children’s Hospital, Capital Medical University. All patients’ parents or guardians signed informed consent forms.

### Conditioning regimen

**Fig. 1:**
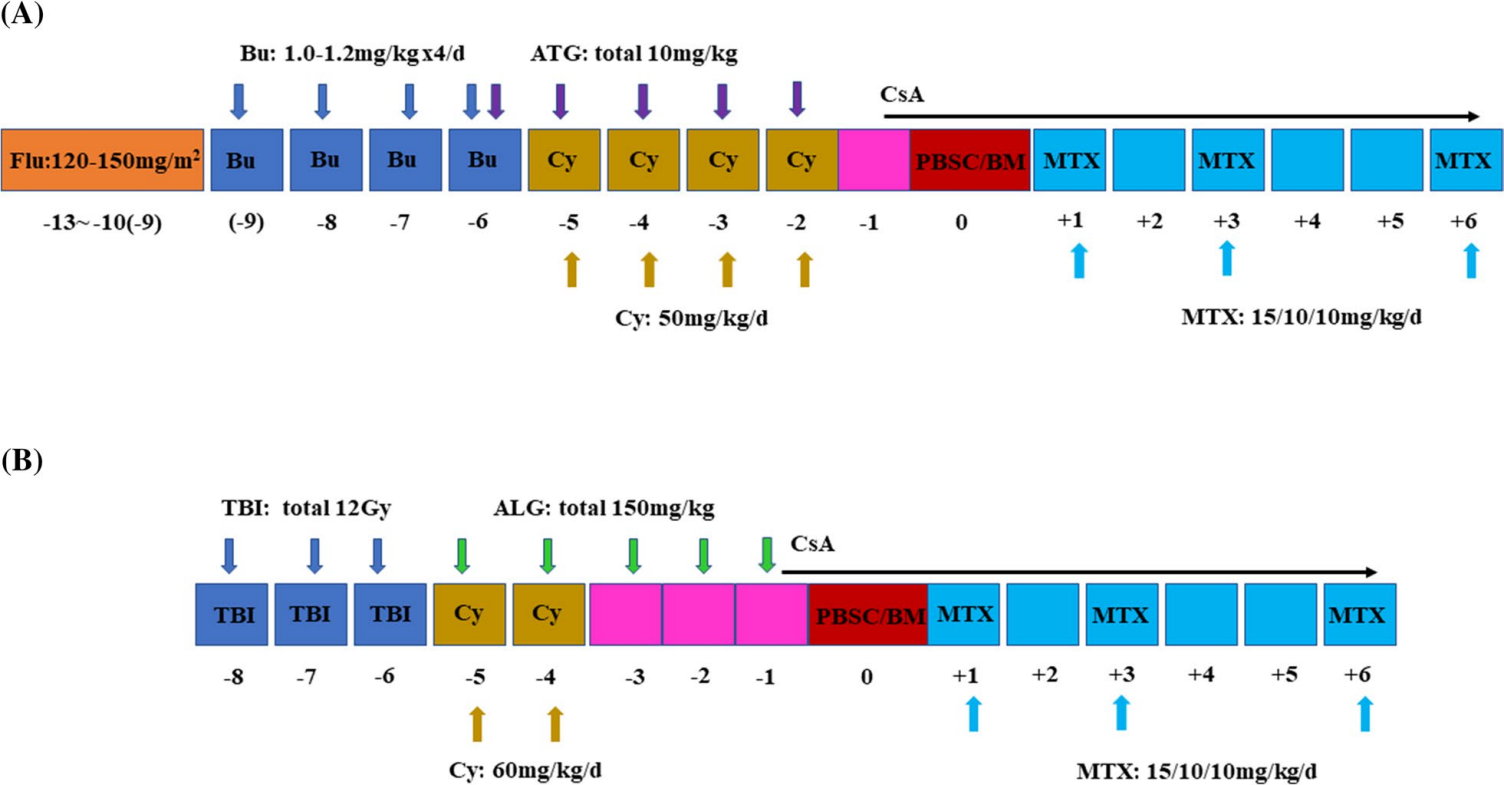
Conditioning regimen for patients with malignant infantile osteopetrosis and intermediate osteopetrosis

All patients received transplants from unmatched related donors and myeloablative conditioning regimens. Fludarabine (Flu) was administered at a dose of 120–150 mg/m^2^, busulfan (Bu) was administered at 16–19.2 mg/kg (weight adapted, due to our technology, most children did not undergo PK testing) and cyclophosphamide (Cy) was administered at 200 mg/kg to the 26 patients. One patient received TBI (12 Gy) and Cy 120 mg/kg as a conditioning regimen. An individualized dose of treatment was used according to the patient’s condition (Fig. 1). Compatibility was defined by HLA-A/B/C/DR/DQ serotypes and high-resolution molecular HLA typing. All donors were injected subcutaneously with granulocyte colony-stimulating factor (5 μg/kg twice daily for 5 days). Mobilized PBSCs and bone marrow were collected on days 5 and 6.

### GVHD prophylaxis and treatment

Graft versus host disease (GVHD) prophylaxis: Acute and chronic graft versus host disease (GVHD) were diagnosed and graded by physicians according to defined criteria [8, 9]. The diagnosis of GVHD mainly depends on the signs and symptoms of patients. All patients received cyclosporine A (CsA) at 5 mg/kg per day from d-1; mycophenolate mofetil (MMF) at 15 mg/m^2^ on d1 and 10 mg/m^2^ on d3, 6, and 11; methotrexate (MTX) and anti-human thymocyte globulin (ATG) at 10 mg/kg or anti-human T lymphocyte porcine immunoglobulin (ALG) at 150 mg/kg to prevent the occurrence of GVHD.

### Engraftment and chimaerism

The evidence of engraftment included increases in peripheral white blood cell and monocyte counts and the presence of mature granulocytes 2–4 weeks after transplantation, which were confirmed by a chimaerism analysis. Chimaerism was detected using routine karyotyping, and DNA fingerprinting of short tandem repeats was conducted on whole blood samples at one month, three months, six months, and twelve months after transplantation. Neutrophil engraftment was defined as the first day of an absolute neutrophil count > 0.5 × 10^9^/L for 3 consecutive days. Platelet engraftment was defined as a platelet count > 50 × 10^9^/L for at least 7 days without transfusion support. Primary graft failure was defined as the absence of donor-derived myeloid cells on day + 30 or reconstitution with autologous cells, and secondary graft failure was defined as the loss of a previously functioning graft, resulting in cytopenia involving at least 2 blood cell lineages and confirmed by the chimaerism analysis when the technique was available [10].

### Supportive care

Thrombotic microangiopathy (TMA) was diagnosed by physicians according to defined overall TMA (O-TMA) criteria reported by Cho et al [11]. Patients were diagnosed with hepatic veno-occlusive disease (HVOD) based on the Seattle criteria, depending on the signs/symptoms of patients and the result of a liver ultrasound [12]. Ursodeoxycholic acid was administered at a dose of 5–7 mg/kg per day po (from d-14) and low molecular weight heparin was administered at a dose of 100 IU/kg per day by subcutaneous injection (from d-10) to prevent the occurrence of HVOD. Phenytoin sodium was administered at a dose of 5 mg/kg per day po (from d 14), followed by gradual tapering for the next 1.5 weeks to prevent epilepsy. G-CSF was subcutaneously injected at a dose of 5 µg/kg per day (from d + 5) to stimulate haematopoiesis. Acyclovir was iv injected at a dose of 30 mg/kg per day (from d0) to prevent viral infection. Viral PCR screening and quantification were performed weekly for cytomegalovirus, Epstein-Barr virus, and adenovirus. All patients were administered antimicrobial prophylaxis for fungi. HSCT recipients were treated in single rooms with laminar airflow systems.

### End points

The primary end point was survival. Death from any cause was considered an event, and surviving patients were censored at the last follow-up. After the transplant, patients were required to return to the hospital once a month for posttransplant evaluations after the first discharge, including routine blood tests, biochemical tests, imaging, and chimaerism analyses. The time of last follow-up was defined as the number of days between the date of transplantation and the last clinic visit.

### Statistical analysis

Statistical analyses were performed using IBM SPSS Statistics 24 software (IBM, USA). Data with a skewed distribution are presented as medians (interquartile ranges). The log-rank test was used to verify overall survival and to compare the survival rates between different groups. P < 0.05 indicated a significant difference.

## Results

### General patient information

Twenty-seven patients with osteopetrosis were enrolled in this study, including 19 males and 8 females. The ratio of males to females was 2.375:1. Among the patients, 23 were diagnosed with MIOP and 4 were diagnosed with IOP. The median age of disease onset was 12 (0.04–72) months. The median age of diagnosis was 8 (0.1–84) months. Twenty-two patients (81.5%) were less than 1 year old at disease onset. The onset age of MIOP was 2 (0.04–29) months and that of IOP was 33 (21–72) months. The median time from diagnosis to transplantation was 4 (1–23) months.

All the children who underwent a bone X-ray examination showed increased density with a reduced/disappeared bone marrow cavity. Ten patients had skull deformities, including two with square heads and one with lost teeth. Two patients had deformed X-shaped legs. Twenty-six patients (96.3%) presented optic nerve damage, and imaging indicated optic canal stenosis. Eighteen patients experienced clinical manifestations, including double nystagmus in 11 patients, abnormal ocular pursuit in 4 patients, exotropia in 2 patients, and blurred vision in 8 patients. Five patients (18.5%) had a hearing impairment. Splenomegaly was observed in 23 children, and hepatomegaly was observed in 26 children. The laboratory examination showed that all patients had anaemia 80 (49–115) g/L (reference range 120–190 g/L), and 21 patients had thrombocytopenia 55 (7–174) × 10^9^/L (reference range 100–400 × 10^9^/L). Eleven patients had liver damage with high levels of ALT and AST, and 26 patients had myocardial damage with high levels of CK-MB 82 (24-1061) U/L (reference range 0–25 U/L). Ten patients had an infection history, and two patients had a bone fracture history.

CLCN7 gene mutations were identified in 6 patients, including 2 compound heterozygous and 4 heterozygous mutations. TCIRG1 gene mutations were detected in 18 patients. The remaining 3 patients did not undergo a genetic analysis (Table 1).

**Table 1:** General information

Total patients	27
Sex (%)	
Male	18 (66.7)
Female	9 (33.3)
Age at transplant (median)	12 months (range: 4–107 months)
Gene (%)	
TCIRG1 compound heterozygous mutation	18 (66.7)
CLCN7 compound heterozygous mutation	2 (7.4)
CLCN7 heterozygous mutation	4 (14.8)
Visual impairment	26/27
Hearing impairment	5/27
Splenomegaly	23/27
Abnormal haemogram	27/27

### Engraftment and chimaerism

One patient failed to receive the same matched unrelated donor transplantation twice and then underwent unmatched related donor transplantation from her father. The remaining 26 patients underwent unmatched related donor transplantation. The median infused mononuclear cell (MNC) count was 23.12 (10.04–51.90) × 10^8^ cells/kg, and the median infused CD34^+^ cell count was 10.22 (5.96–24.88) × 10^6^ cells/kg. The graft source and conditioning regimen are shown in Table 2.

**Table 2:** Engraftment and GVHD

Overall survival (%)	73.9%
Engraftment source	
Father	19 (70.4)
Mother	8 (29.6)
HLA-matched	
5/10 (3/6)	18 (66.7)
6/10	2 (7.4)
7/10	4 (14.8)
5/6	2 (7.4)
9/10	1 (3.7)
Conditioning regimen	
TBI (12) + Cy (120)	1
Flu (120–150) + Bu (16–19.2) + Cy (200)	26
Blood type	
Matched	12
Mismatched	15
Stem cells infused	
MNC × 10^8^/kg	23.12 (10.04–51.90)
CD34^+^ × 10^6^/kg	10.22 (5.96–24.88)
aGVHD (%)	21/27 (77.8)
Stage I–II	20
Stage III	1
Stage IV	0
cGVHD (%)	11/27 (40.7)
Infection	
CMV infection (dead/alive)	0/15
EBV infection, reactivated	10
Bacterial	12
Haemorrhagic cystitis	3
HVOD (%)	5 (18.5)
Engraftment failure	0

TBI, total body irradiation; Cy, cyclophosphamide; Flu, fludarabine; Bu, busulfan; GVHD, graft versus host disease; HVOD, hepatic veno-occlusive disease

**Fig. 2:**
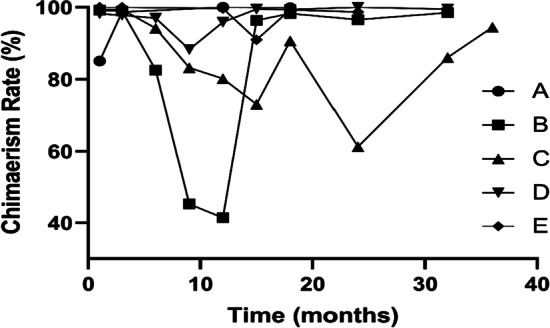
The donor chimaerism rate of five patients with a mixed donor type. Two patients (**A** and **B**) presented a mixed donor type in the early stage after transplantation, and the lowest chimaerism rates were 45.2% and 85.1%, respectively. The type changed to the full donor type after donor CD34^+^ cells were reinfused. However, three patients (**C**–**E**) exhibited the mixed donor type 6 months after transplantation that changed to the full donor type after donor CD34^+^ cells were reinfused or a donor lymphocyte transfusion

Twenty-six patients experienced successful neutrophil engraftment, and the median time of engraftment was 25 (10–37) days. Twenty-three patients experienced successful platelet engraftment, and the median time of engraftment was 43 (10–155) days. Donor chimaerism was 92.6% at day 30 posttransplantation. Two patients (A and B) presented a mixed donor type in the early stage after transplantation, and the lowest chimaerism rates were 45.2% and 85.1%, respectively. This type changed to the full donor type after donor CD34^+^ cells were reinfused. However, three patients (C, D and E) exhibited a mixed donor type 6 months after transplantation that changed to the full donor type again after donor CD34^+^ cells were reinfused or a donor lymphocyte transfusion (Fig. 2).

### GVHD and transplant-related morbidity

In our study, 21 (77.8%) patients suffered acute GVHD (aGVHD), twenty of whom were graded as stage I-III and one of whom was graded as stage III. The most common location of aGVHD was the skin (n = 19), with a red haemorrhagic rash predominating and without sclerosis. The remaining 3 aGVHD cases involved the gastrointestinal tract. Chronic GVHD (cGVHD) was observed in 11 patients (40.7%). Among them, ten presented skin involvement, including 3 cases that were widely distributed throughout the body, whereas the remaining cases were locally limited. The other patient presented with gastrointestinal tract involvement along with diarrhoea. After treatment with corticosteroids and tacrolimus, the GVHD of the children improved, drugs were withdrawn from some patients, and no child died of GVHD.

In terms of transplant-related morbidity, 15 patients (55.6%) had a CMV infection, including one case of CMV pneumonia. All patients’ plasma CMV-DNA tests became negative after antiviral therapy. Ten patients had an EBV infection or reactivation. Twelve patients had a bacterial infection, including 10 cases of pneumonia and 2 cases of septicaemia. Hypercalcaemia occurred in 4 patients with a median serum calcium level of 2.95 (2.85–2.99) mmol/L, which was improved after proper treatment. Four patients experienced autoimmune haemolytic anaemia, and 3 patients experienced haemorrhagic cystitis. Five patients had HVOD, and 1 patient had transplantation-related thrombotic microangiopathy.

### Follow-up and survival

**Fig. 3:**
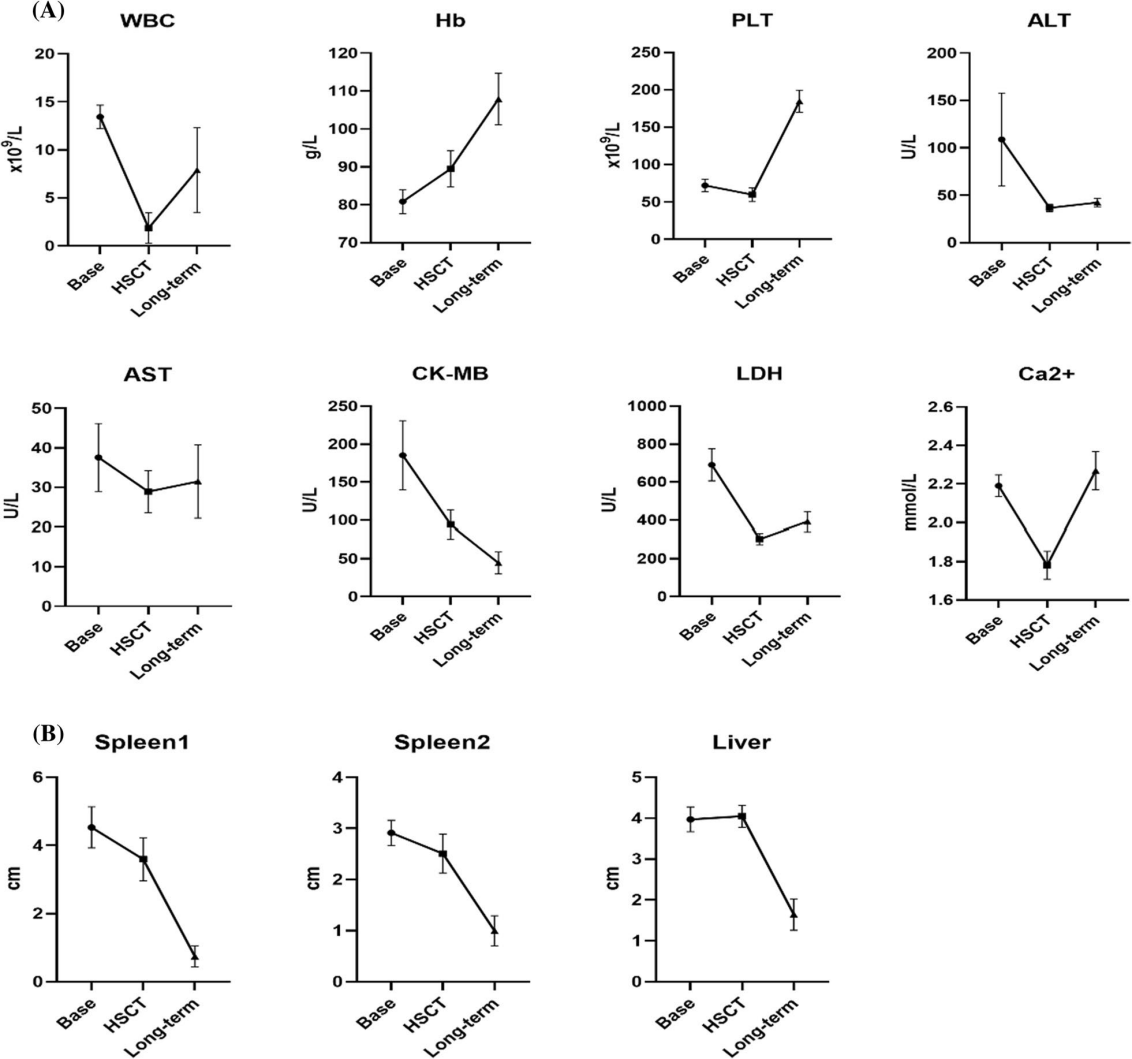
Clinical characteristics of children with MIOP/IOP and progression after treatment (**A**, **B**), “Base” indicates detection at diagnosis; “HSCT” indicates detection at HSCT; “Long-term” indicates detection at the end of follow-up. “Spleen 1” represents the lower margin of the spleen under the subcostal margin, which was measured using abdominal ultrasound. “Spleen 2” represents the thickness of the spleen, which was measured using abdominal ultrasound. “Liver” represents the lower margin of the liver under the subcostal margin, which was measured using abdominal ultrasound. WBC: white blood cell; Hb: haemoglobin; PLT: platelet; AST aspartate aminotransferase; ALT alanine aminotransferase; CK-MB: creatine kinase-MB; LDH lactic dehydrogenase; Ca^2+^: calcium

**Fig. 4:**
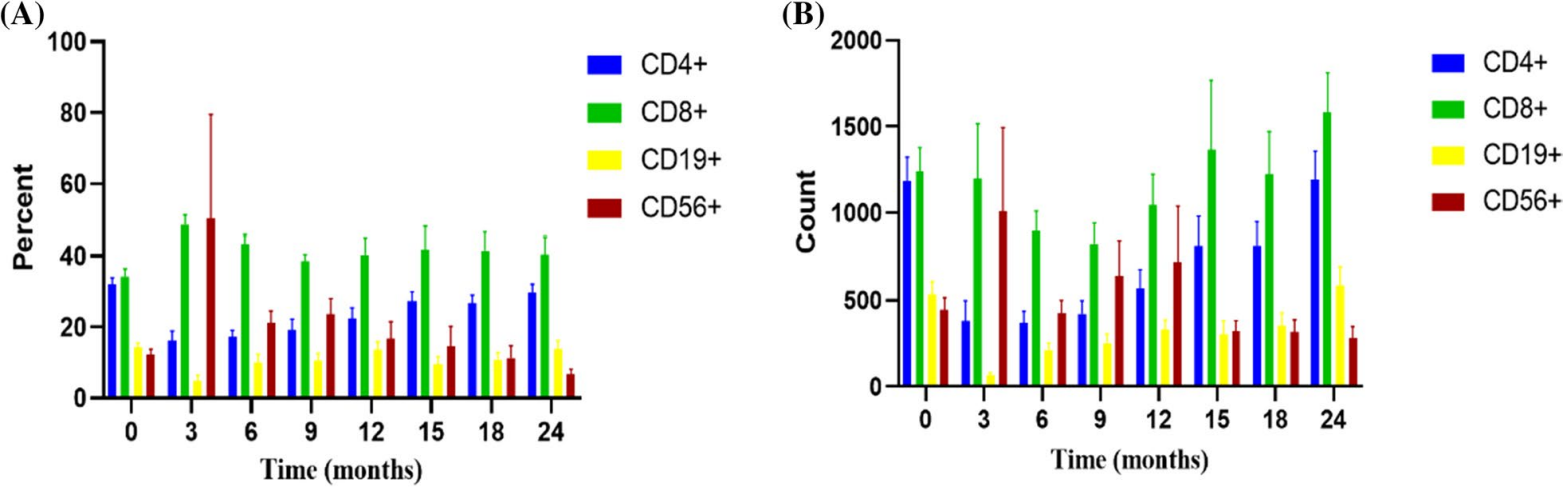
Changes in CD subtypes after HSCT. (**A**) Percent and (**B**) absolute count

**Fig. 5:**
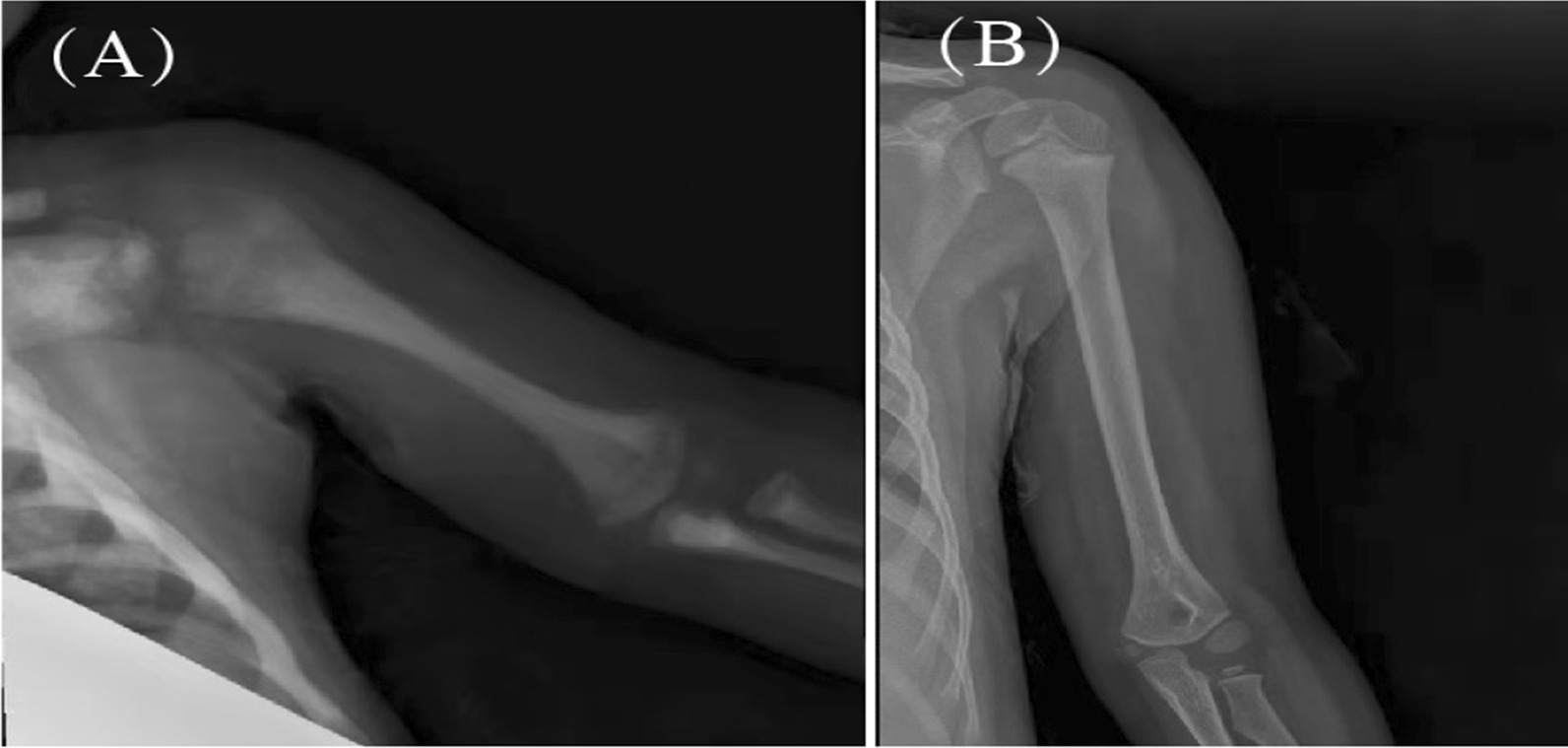
The bone density of the left humerus increased unevenly, and the bone cortex thickened before transplantation (**A**). Four years after transplantation, the bone mineral density decreased, and the bone marrow cavity was clear (**B**)

The last follow-up was November 1, 2020, and the median follow-up time was 55.2 (0.3–126.2) months. No patients were lost to follow-up. Among the enrolled patients, 20 patients survived and 7 patients died. All deaths occurred within 2 months after the transplant. Causes of death were pulmonary haemorrhage (42.8%), severe pneumonia after transplant (28.6%), heart failure secondary to autoimmune haemolytic anaemia (14.3%), and gastrointestinal bleeding and multiple organ failure secondary to HVOD (14.3%). Among all survivors, no patients experienced graft failure or rejection. The disease was controlled, and the haematopoietic function was restored [WBC 6.60 (5.24–22.32) × 10^9^/L, Hb 129 (122–146) g/L, and PLT 210 (126–308) × 10^9^/L]. The myocardial damage in most patients also recovered. The size of the liver and spleen returned to normal (Fig. 3). Sixteen patients were continuously monitored for changes in CD subclasses in our hospital, and the results are shown in Fig. 4. The bone X-ray examination at 6 months after transplantation showed that the bone mineral density was lower than before surgery, and the bone marrow cavity gradually formed 9–12 months after transplantation, suggesting bone remodelling (Fig. 5). The visual acuity of 17 children was monitored continuously in our hospital. The eye condition was assessed using a fundus examination, electroretinogram, visual evoked potential, flash visual evoked potential and head CT. Among them, one patient returned to normal visual acuity, three patients exhibited improved optic canal stenosis and optic nerve compression. The visual acuity of the other 3 cases was not significantly altered. Hearing was monitored using pure tone audiometry and auditory brainstem response tests. The hearing condition of the five patients who had a hearing impairment improved but did not return to normal.

**Fig. 6:**
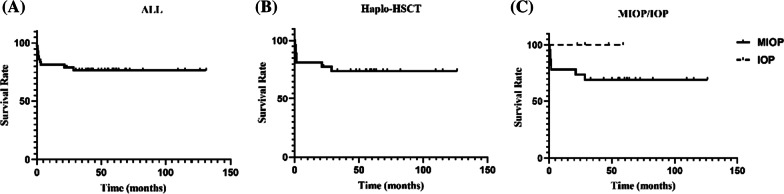
The 5-year overall survival (OS) rates of all patients with osteopetrosis who underwent HSCT was 76.7% (**A**). The 5-year overall survival (OS) rate was 73.9% in patients who underwent haplo-HSCT (**B**). A comparison of patients with MIOP and IOP did not reveal a statistically significant difference (69.6% vs. 100.0%, P = 0.2444) (**C**). ALL: all patients

The 5-year overall survival (OS) was 73.9% in our cohort of 27 patients who underwent haplo-HSCT. The 5-year OS of all patients with osteopetrosis who underwent HSCT (including matched unrelated donors, matched related donors and other donors) was 76.7% in the same period. A statistically significant difference was not observed between patients with MIOP and IOP (69.6% vs. 100.0%, P = 0.2444) (Fig. 6). According to the source of haematopoietic stem cells, we divided the patients into stem cells from the father and mother, and no statistically significant difference was observed in 5-year OS between these groups (68.4% vs. 87.5%, P = 0.273). Patients were divided into two groups according to whether the donor and patients had the same blood type, and no statistically significant difference in 5-year OS was observed between groups (66.7% vs. 79.4%, P = 0.373). Based on the log-rank analysis, statistically significant associations between the age at onset, age at transplantation and infused CD34^+^ cell count and 5-year OS were not observed.

## Discussion

Osteopetrosis is a disease caused by the differentiation, development, or dysfunction of osteoclasts. The balance between osteoclasts and osteoblasts is very important for maintaining bone homeostasis. When osteoclasts are dysfunctional, a series of clinical manifestations may arise, such as short stature, fractures, osteomyelitis, hypocalcaemia, convulsions, and neurological lesions [13]. Defective osteoclast activity can be caused by mutations in genes affecting osteoclast development (such as RANK and RANKL) and function (such as TCIRG1, SNX10, CLCN7, and OSTM1). Biallelic mutations in these genes will lead to malignant infantile osteopetrosis (MIOP). This subtype has severe clinical manifestations and a poor prognosis. The onset age is early, generally in the first year after birth. Autosomal dominant osteopetrosis is caused by monoallelic mutations in the CLCN7 gene, which is called IOP. The onset age of this type is relatively late, and the progression of the disease is slow [14]. In the present study, 22 patients (81.5%) were less than 1 year old at the time of onset, the age of onset of MIOP was 2 (0.04–29) months, and the age of onset of IOP was 33 (21–72) months.

Because osteoclasts are myeloid cells and allogeneic haematopoietic stem cell transplantation can provide osteoclasts for patients, allogeneic HSCT is the only effective treatment for MIOP at present [15]. Driessen et al. [5] reported 122 patients who underwent allogeneic HSCT treatment for MIOP and found that the overall successful engraftment rate was 77%. The higher failure rate of transplantation was mainly related to dysfunction of bone haematopoietic capacity, spleen retention or destruction of stem cells and HLA mismatch. This article also found that the 5-year EFS of HLA-matched patients was significantly higher than that of HLA-mismatched patients (73% vs. 24%). Natsheh et al. [16] reported 38 patients who underwent HSCT for MIOP, including 36 HLA-matched patients, 1 haploidentical transplant recipient and 1 T cell-depleted (TCD) in vitro transplant recipient. The 5-year OS was 84%. Orchard et al. [17] reported the long-term survival of 193 patients with MIOP after transplantation. Eighty-nine patients underwent transplantation using grafts from HLA-matched siblings and 104 patients received transplants from HLA-mismatched donors (including 25 patients with transplants from mismatched sibling donors and 79 patients with transplants from mismatched unrelated donors). The conditioning regimen was Bu and Cy. The 5- and 10-year probabilities of survival were 62% and 62% after HLA-matched sibling transplantation and 42% and 39% after mismatched donor transplantation. Graft failure was the most common cause of death. Bahr et al. [18] reported 3 patients with MIOP who were treated with Haplo-HSCT. With the posttransplantation cyclophosphamide regimen, only 1 patient survived. Stepensky et al. [14] reported that 6 of 7 children with IOP survived after receiving HSCT, and 1 died of CMV infection and pulmonary hypertension after haplo-HSCT. In summary, previous research indicated that the first choice for MIOP treatment is an HLA-matched sibling donor for HSCT, and that haplo-HSCT is not a suitable treatment for MIOP and IOP. Graft failure was the main factor affecting the treatment of MIOP and IOP with haplo-HSCT.

However, in China, HLA-matched sibling donors are difficult to obtain for HSCT. The time for seeking matched unrelated donors is long, and the success rate is not high. Parents who are carriers can become potential donors, which can reduce the waiting time and complications. Torres et al. [10] reported 2 patients who were treated with in vitro TCD (purified CD34^+^ stem cells) and Bu, Flu combined with ATG as a conditioning regimen. One patient survived, and 1 patient died of graft failure. Driessen et al. [5] reported 37 patients with MIOP who were treated with TCD in vitro, with an overall survival rate of 34%, and the main cause of death was severe infection. In contrast to previous research, in this study, 27 patients received haplo-HSCT treatment. Twenty patients survived, and the 5-year OS rate was 73.9%. This retrospective observational study showed that the in vivo T removal regimen achieved a more successful engraftment rate than the in vitro T removal regimen, which was approximately or even higher than previous reports of HLA-matched HSCT. In terms of the conditioning regimen, previous research found that the 5-year OS in the fludarabine group was higher than that in the group treated without fludarabine (96% vs. 58%). Our research indicated that the myeloablative conditioning regimen was beneficial for stem cell engraftment. In the present study, the 30-day neutrophil engraftment rate was greater than 95%. Although the platelet engraftment time was later, the 120-day engraftment rate was more than 60%. Faster stem cell engraftment was beneficial to haematopoietic recovery, and it was more convenient for a donor lymphocyte transfusion due to poor engraftment. In addition, compared with umbilical cord blood HSCT, haplo-HSCT had another advantage. A previous report described 51 patients who received umbilical cord blood HSCT for MIOP, and the 6-year OS rate was 43%. Most of the patients died of engraftment failure [19]. Compared with cord blood HSCT, mobilized bone marrow and peripheral blood stem cells may provide more CD34^+^ cells for patients, which would promote engraftment.

Our study adopted the “Beijing Protocol”, which included the myeloablative conditioning regimen (Flu + Bu + Cy), the in vivo TCD regimen (ATG/ALG), and stem cells collected from mobilized bone marrow and peripheral blood [20]. All these measures worked together to reduce the occurrence of severe GVHD. Acute GVHD stage I-II was observed in 20 patients, stage III disease occurred in 1 patient and no patients had stage IV GVHD. Chronic GVHD was observed in 11 patients, ten of whom had skin involvement, and 7 of whom had a locally limited disease. The condition of GVHD was improved after using hormones and other anti-GVHD drugs, such as tacrolimus, a CD25 monoclonal antibody and ruxolitinib. The data indicated that the incidence of GVHD was high but mild and was effectively controlled by the appropriate treatment. GVHD would not affect the survival of patients. Kapelushnik et al. [21] found that patients who died of acute respiratory distress syndrome or pulmonary haemorrhage after transplantation may have pulmonary arterial hypertension. According to another study, ATP6i (TCIRG1) mutations lead to pulmonary arterial hypertension [22]. In our study, 3 children who died of pulmonary haemorrhage all had TCIRG1 mutations, but pulmonary haemorrhage and pulmonary arterial hypertension may also be related to the aggravation of pulmonary symptoms caused by high-dose chemotherapy and massive fluid infusion. Patients who experienced these severe toxicities all developed pneumonia before transplantation. Therefore, the poor condition before HSCT might be related to severe toxicities. The myeloablative conditioning regimen led to a higher engraftment rate, but the toxicity was also relatively high, especially the incidence of HVOD [23]. In our study, the early addition of ursodeoxycholic acid, low molecular weight heparin calcium and defibrinylate significantly reduced the HVOD death rate. Hypercalcaemia was also a serious complication after transplantation, mainly because of the short-term release of large amounts of intraosseous calcium from bone into the blood [24]. In the present study, only 4 patients experienced hypercalcaemia, and the elevated serum calcium levels were not significant. This finding might be because the donor was also a carrier, and the function of osteoclasts was lower than normal. A large amount of calcium is unable to easily enter the blood in a short period and be compensated by the body.

This retrospective observational study also observed significant bone remodelling after haplo-HSCT, and the curative effect was lasting and stable. However, other complications, especially blindness and deafness caused by nervous system involvement, were not significantly improved after transplantation. A previous study showed that only 7% of eyesight was restored, and vision problems still progressed in 25% of patients. In our study, only one patient’s visual acuity returned to normal, and the visual acuity of 13 patients was improved, which may be related to the older age at the time of diagnosis and the involvement of the nervous system before HSCT. Although our study did not find a relationship between the age at diagnosis, age at transplantation and prognosis, early diagnosis and transplantation are very to improve the quality of life and reduce the rate of disability.

In this study, 66.7% of the patients had TCIRG1 gene mutations (recessive inheritance), and 22.2% had CLCN7 gene mutations (recessive and dominant inheritance). Currently, controversy still exists regarding which patients are suitable for HSCT. For patients with TCIRG1 mutations, once diagnosed, they should undergo HSCT as soon as possible. However, for some patients with CLCN7 and OSTM1 gene mutations, previous articles showed that central involvement was irreversible and might be further aggravated after HSCT, and thus transplantation was not recommended [6, 25]. However, 6 patients with CLCN7 gene mutations were included in this group, including 2 patients with compound heterozygous mutations and 4 patients with heterozygous mutations (without neurodegeneration). In the present study, the clinical symptoms were significantly improved and no aggravation of nervous system involvement was observed after transplantation. Therefore, we postulate that these patients should still undergo HSCT if the disease occurs early with typical clinical manifestations [13, 26]. Furthermore, if patients have RANKL gene mutations, HSCT is not recommended because abnormalities in the gene may impede osteoclast maturation [27].

## Conclusions

Based on the results described above, haplo-HSCT is an effective and feasible treatment for MIOP and IOP. The incidence of GVHD was high but mild and was effectively controlled after appropriate treatment. However, the symptoms of nervous system involvement did not readily recover, and thus the early identification and diagnosis of MIOP and IOP are the key to improving the prognosis. If no sibling and unrelated matched donors are available, a haploidentical related donor is also an important choice. The number of patients in this study was small, and the follow-up time must be increased to expand the analysis of the prognosis of patients with MIOP and IOP, especially the growth and intellectual development of children. The mortality rate of pulmonary haemorrhage and severe pneumonia in patients was high, which might be related to the intensity of the conditioning regimen, and thus further adjustments to the dose of the conditioning regimen are needed. The occurrence of aGVHD might be related to the larger number of infused cells, which also requires further adjustment.

## Data Availability

The data that support the findings of this study are available on request from the corresponding author.

## Abbreviations

MIOP: Malignant infantile osteopetrosis
IOP: Intermediate osteopetrosis
haplo-HSCT: Haploidentical allogeneic haematopoietic stem cell transplantation
Flu: Fludarabine
Bu: Busulfan
Cy: Cyclophosphamide
GVHD: Graft versus host disease
CsA: Cyclosporin A
MMF: Mycophenolate mofetil
ALG: Anti-human T lymphocyte porcine immunoglobulin
ATG: Anti-human thymus globulin
HVOD: Hepatic veno-occlusive disease
OS: Overall survival
TCD: T cell-depleted
PTC: Posttransplantation cyclophosphamide
